# A Novel Glycosylated Ferulic Acid Conjugate: Synthesis, Antioxidative Neuroprotection Activities In Vitro, and Alleviation of Cerebral Ischemia–Reperfusion Injury (CIRI) In Vivo

**DOI:** 10.3390/antiox14080953

**Published:** 2025-08-03

**Authors:** Jian Chen, Yongjun Yuan, Litao Tong, Manyou Yu, Yongqing Zhu, Qingqing Liu, Junling Deng, Fengzhang Wang, Zhuoya Xiang, Chen Xia

**Affiliations:** 1 Institute of Agro-Products Processing Science and Technology (Institute of Food Nutrition and Health), Sichuan Academy of Agricultural Sciences, Chengdu 610066, China; manyou_yu@scsaas.cn (M.Y.); zhuyongqing68@sina.com (Y.Z.); junlinr@scsaas.cn (J.D.); xiangzy@scsaas.cn (Z.X.); 2 School of Food and Bioengineering, Xihua University, Chengdu 610039, China; yyja9791@sina.com (Y.Y.); liuqingqing@xhu.edu.cn (Q.L.); 3 Institute of Food Science and Technology, Chinese Academy of Agricultural Sciences/Key Laboratory of Agro-Products Processing, Ministry of Agriculture, Beijing 100193, China; tonglitao@caas.cn (L.T.); wangfengzhang@caas.cn (F.W.)

**Keywords:** ferulic acid, glycosylation conjugate, antioxidant, neuroprotection, CIRI

## Abstract

Antioxidative neuroprotection is effective at preventing ischemic stroke (IS). Ferulic acid (FA) offers benefits in the treatment of many diseases, mostly due to its antioxidant activities. In this study, a glycosylated ferulic acid conjugate (FA-Glu), with 1,2,3-triazole as a linker and bioisostere between glucose at the C6 position and FA at the C4 position, was designed and synthesized. The hydrophilicity and chemical stability of FA-Glu were tested. FA-Glu’s protection against DNA oxidative cleavage was tested using pBR322 plasmid DNA under the Fenton reaction. The cytotoxicity of FA-Glu was examined via the PC12 cell and bEnd.3 cell tests. Antioxidative neuroprotection was evaluated, in vitro, via a H_2_O_2_-induced PC12 cell test, measuring cell viability and ROS levels. Antioxidative alleviation of cerebral ischemia–reperfusion injury (CIRI), in vivo*,* was evaluated using a rat middle cerebral artery occlusion (MCAO) model. The results indicated that FA-Glu was water-soluble (LogP −1.16 ± 0.01) and chemically stable. FA-Glu prevented pBR322 plasmid DNA cleavage induced via •OH radicals (SC% 88.00%). It was a non-toxic agent based on PC12 cell and bEnd.3 cell tests results. FA-Glu significantly protected against H_2_O_2_-induced oxidative damage in the PC12 cell (cell viability 88.12%, 100 μM) and inhibited excessive cell ROS generation (45.67% at 100 μM). FA-Glu significantly reduced the infarcted brain areas measured using TTC stain observation, quantification (FA-Glu 21.79%, FA 28.49%, I/R model 43.42%), and H&E stain histological observation. It sharply reduced the MDA level (3.26 nmol/mg protein) and significantly increased the GSH level (139.6 nmol/mg protein) and SOD level (265.19 U/mg protein). With superior performance to FA, FA-Glu is a safe agent with effective antioxidative DNA and neuronal protective actions and an ability to alleviate CIRI, which should help in the prevention of IS.

## 1. Introduction

Stroke is the second leading cause of death worldwide. Ischemic stroke (IS) accounts for more than 87 % of all recorded strokes [1]. The current primary treatment protocol for IS includes either thrombolysis or thrombectomy to restore cerebral blood flow. Although reperfusion of blood flow can preserve some of the dying brain tissue [2], it triggers a cascade of pathophysiological reactions, such as calcium dysregulation, oxidative stress, and inflammation. These reactions worsen brain dysfunction and cause structural tissue damage, namely inducing cerebral ischemia–reperfusion injury (CIRI) [3]. Patients are at risk of CIRI due to the narrow therapeutic time window [4]. The neurological impairments caused by CIRI are lethal and disabling. Reoxygenation of ischemic tissues during reperfusion produces excessive reactive oxygen species (ROS), which cause cytotoxicity through the oxidation of lipids, proteins, and DNA, thereby damaging the brain’s cellular integrity and function, leading to either reversible or permanent neurological deficits [5,6]. Overproduction of ROS overwhelms cellular defenses and leads to the development of CIRI. Therefore, the exogenous supplementation of antioxidants with ROS scavenging activity could be a potential strategy to alleviate CIRI.

Ferulic acid (FA), a phenolic compound of cinnamic acid, is abundant in cereals, fruits, vegetables [7,8], and Chinese herbal medicines, such as reed root, angelica, and wild ferula. FA is well known for its antioxidant and anti-inflammatory activities, which have therapeutic benefits in diseases such as neurodegenerative disease, diabetes mellitus, cancer, hypertension, atherosclerosis, and obesity [9,10]. It is commonly recognized that FA contributes to antioxidant effects by donating hydrogen atoms to free radicals. FA possesses potent antioxidant activity because it transforms into a resonant-stable radical after losing its hydrogen atom [11,12,13]. FA is also an inhibitor of the enzyme that catalyzes the production of free radicals and enhances the enzyme activity of the scavenger [14]; FA can reduce malondialdehyde (MDA) content, increase SOD activity and the glutathione (GSH) content, and inhibit the formation of ROS [15]. FA has been widely used in the pharmaceutical, food, cosmetic, and feed industries. However, there are still some problems in the application of FA. In particular, FA has poor water solubility (0.8 g/L) and unstable physicochemical properties [16]. In addition, there is limited research on improving the bioavailability and organ and/or tissue targeting ability of FA through chemical modifications. The glycosylation of a molecule usually modifies its hydrophilicity, bioactivity, bioavailability, stability, and chemical properties [14,17]. FA naturally exists in plant cell walls, where it links to Arabinoxylan via an ester bond and Lignin via an ether bond at the 4-hydroxy position, forming a complex with antimicrobial properties in the plant. A previous study showed that the glycosylation of hydroxycinnamic acids can increase their bioavailability [18]. The glycosylation of FA via glucose produced 4-*O*-β-D-glucopyranosyl FA. This was achieved via chemical synthesis [18] or the biosynthesis of ferulic acid glycosides in plant tissue cultures [19], and was also efficiently synthesized via a microbe-derived glycosyltransferase catalysis [17]. 4-*O*-β-D-Glucopyranosyl FA glycosylation was accomplished between the C1 hemiacetal of glucose and the 4-hydroxyl position of FA. In this work, a novel glycosylation FA conjugate (FA-Glu) was designed and prepared for the purpose of improving the water solubility, stability, antioxidant activities, and brain delivery of precursor FAs, as well as its neuroprotection effect against ischemic stroke (IS).

## 2. Materials and Methods

### 2.1. Materials and Equipment

All liquid reagents were initially distilled unless otherwise stated. All unspecified reagents were from commercial sources. TLC was conducted with precoated silica gel GF254 (0.2 mm, Kelong Chemical, Chengdu, China), similarly to column chromatography (silica gel, 200–300 mesh). ^1^H NMR spectra were recorded at 400 MHz (Varian, Palo Alto, CA, USA), and ^13^C NMR spectra were recorded at 101 MHz (Varian, USA). Preparative HPLC was performed using Agilent Technologies 1260 Infinity II, USA. Fluorescence images were captured using a laser scanning confocal microscope (LSM800, Carl Zeiss AG, Jena, Germany). FA was obtained from the Sichuan Academy of Agricultural Sciences (Chengdu, China) and supercoiled pBR322 plasmid DNA (4361 bp, MW. 2.83 MDa) was obtained from Takara Bio Inc (Beijing, China). 3-(4, 5-Dimethylthiazol-2-yl)-2,5-diphenyltetrazolium bromide (MTT), the total reactive oxygen species (ROS) detection kit, the total superoxide dismutase (SOD) assay kit, the lipid peroxidation MDA assay kit, 2,3,5-triphenyltetrazolium chloride (TTC), and hematoxylin and eosin (H&E) staining solution were purchased from Beyotime Institute Biotechnology (Haimen, China). The reduced glutathione (GSH) assay kit was obtained from Nanjing Jiancheng Bioengineering Institute (Nanjing, China), and mouse brain microvascular endothelial cells (bEnd.3 cells) and rat adrenal pheochromocytoma cells (PC12 cells) were purchased from the Chinese Academy of Sciences Cell Bank.

### 2.2. Animals

Adult male Sprague-Dawley (SD) rats (age 8–9 weeks, weight 230 ± 20 g) were obtained from Chengdu Dashuo Experimental Animal Co., Ltd., Certificate Number SCXK[chuan]2024-0185 (Chengdu, China). Animals were housed under standard conditions (temperature 23 ± 0.5 °C, humidity 50–60%, a 12 h light/dark cycle), with free access to feed and water. The rats underwent a 7-day acclimation period before the experiment. Prior to drug administration, all rats were starved overnight, while retaining free access to drinking water. The animal experiments were approved by the Animal Ethics Committee of Sichuan University (Approval No. K2022006, 28 March 2022).

### 2.3. Synthesis of FA–Glucose Conjugate

#### 2.3.1. Synthesis of (*E*)-3-(3-Methoxy-4-(prop-2-yn-1-yloxy)phenyl)acrylic Acid (Compound **1**)

**Scheme 1:**
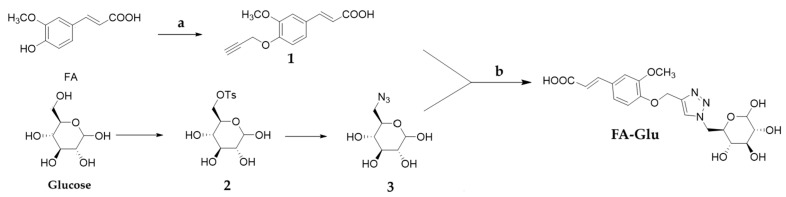
Synthesis route of FA-Glu. Reagents and conditions: (**a**) 3-bromopropyne, K_2_CO_3_, acetone, 60 °C, 12 h; (**b**) Cu_2_SO_4_, NaAs (sodium ascorbate), THF:tBuOH:H_2_O = 2:1:1, r.t.

The reaction route of FA-Glu synthesis is shown in Scheme 1. Starting from glucose, compounds **2** and **3** were prepared according to the procedures of our previous publication [20].

For the preparation of compound **1**, FA (3.0 g, 0.015 mol), 3-bromopropyne (2.21 g, 0.018 mol), and potassium carbonate (2.14 g, 0.015 mol) were dissolved in 40 mL of acetone under nitrogen protection. The reaction mixture was stirred for 12 h at 60 °C. TLC was used to monitor the reaction till its completion. Then, the reaction mixture was extracted 3 times using ethyl acetate (EA). The organic layers were combined and evaporated under reduced pressure, and the residue was purified using flash column chromatography (petroleum ether PE:EA = 4:1) to obtain 1.3 g of compound **1**, which was a white solid with a yield of 37.3%.

#### 2.3.2. Synthesis of FA-Glu

Compound **1** (0.93 g, 0.004 mol), compound **3** (925 mg, 0.005 mol), and cuprous sulfate (Cu_2_SO_4_) (231 mg, 0.001 mol) were dissolved in a mixed solution (THF:tBuOH:H_2_O = 2:1:1, 50 mL) under argon. In total, 273 mg of sodium ascorbate (NaAs) was taken to prepare a 0.1 mM solution, which was then added to the reaction mixture drop by drop at room temperature before stirring overnight. TLC was used to monitor the reaction until completion. The solvents were evaporated under reduced pressure. The residue was first purified using flash column chromatography, followed by further purification using preparative HPLC (column: Shim-pack GIST C18, 5 μm, 20 × 250 mm, Shimadzu, Nakagyo-ku, Kyoto, Japan; eluent: methanol/H_2_O = 1/1; flow rate: 5 mL/min; detection: UV absorbance at 254 nm, column temperature 25 °C). A light-yellow powder product (787 mg) was obtained with a yield of 45%. HPLC purity was 97.35% and Rt = 6.929 min and 7.299 min.

### 2.4. Test of LogP Value

The LogP value was tested to evaluate the hydrophilicity of FA and FA-Glu. A saturated mixture of n-octanol and water was prepared (1:1 *v/v*) and placed in a shaker at 100 rpm under 37 °C for 24 h. A water-saturated n-octanol solution and n-octanol-saturated aqueous solution were obtained after standing overnight. FA and FA-Glu were dissolved in the mixture and left to stand for 2 h after vigorous shaking, respectively. Then, 200 μL of the upper and lower layer was taken for HPLC quantitative detection at 254 nm (eluent methanol/water = 1:1, Gemini 5 μm C18 110 A column, Schimadzu-HPLC, Tokyo, Japan). The LogP value was calculated via the formula LogP = Log (Co/Cw), where Co represents the concentration in the n-octanol layer, and Cw represents the concentration in the water layer.

### 2.5. Test of Stability

FA and FA-Glu were mixed with phosphate-buffer saline (PBS) (pH 7.4), and slowly shaken at 37 °C (45 rpm), respectively. Then, 20 μL of the samples were pipetted out to test the compound content using HPLC at predetermined time points (0 h, 1 h, 2 h, 4 h, 8 h, 12 h, 24 h, and 48 h). The stability was evaluated using the concentration percentage at all time points (concentration% = C_t_/C_0_ × 100%), where C_0_ represents the compound content at 0 h, C_t_ represents the compound content at 1 h, 2 h, 4 h, 8 h, 12 h, 24 h, and 48 h, respectively.

### 2.6. Determination of Preventive Activity Against Oxidative Damage in pBR322 Plasmid DNA

To evaluate the protective effect of FA and FA-Glu against H_2_O_2_-induced oxidative damage to DNA, a slightly modified method described by Xu et al. [21] was employed. In brief, the reaction mixture (15 μL) containing 5 μL of PBS (10 mM, pH 7.4), 1 μL of supercoiled pBR322 plasmid DNA (0.5 μg), 5 μL of 1 mM sample solution, 2 μL of 1 mM FeSO_4_, and 2 μL of 1 mM H_2_O_2_, was incubated at 37 °C for 30 min. After incubation, 2 μL of a loading buffer containing 50% glycerol (*v/v*), 40 mM EDTA, and 0.05% bromophenol blue was added to terminate the reaction. The reaction mixture was electrophoresed (HT-subo1 Agarose Electrophoresis Instrument, Hongtaojiye, Beijing, China) on 1% agarose gel containing 0.1% GelstainRed (UElandy, Suzhou, China) in a Tris/acetate/EDTA gel buffer for 65 min (50 V). The DNA in the gel was visualized and photographed under ultraviolet light. Protection efficacy was calculated as the percentage of supercoiled pBR322 plasmid DNA retained in the presence of samples (DNA + PBS + FeSO_4_ + H_2_O_2_ + Sample) relative to untreated plasmid DNA control (DNA + PBS). Trolox (1 μM) was used as a positive control (DNA + PBS + FeSO_4_ + H_2_O_2_ + Trolox).

### 2.7. Cytotoxicity Test of FA-Glu

The cell viabilities of PC12 and bEnd.3 were assessed after exposure to FA and FA-Glu using the MTT assay, respectively. PC12 and bEnd.3 cells in their logarithmic growth phase were seeded in 96-well plates at a density of 2 × 10^3^ per well and incubated for 24 h. FA and FA-Glu were added into each well with final concentrations ranging from 2.5 to 160 μM for 24 h of incubation, respectively. The medium was replaced with 200 μL MTT solution (5.0 mg/mL in PBS) and incubated for another 4 h. Finally, 150 μL of DMSO was added to dissolve the formazan crystallization, and the cytotoxicity was measured using an MTT assay. The absorbance was measured at 490 nm on an automatic microplate spectrophotometer.

### 2.8. Evaluation of FA-Glu Neuroprotective Effect In Vitro

#### 2.8.1. Antioxidative Protection to H_2_O_2_-Induced Injury on PC12 Cells

The PC12 cells were seeded in 96-well plates at a density of 1 × 10^4^ per well and incubated for 24 h. Then, FA and FA-Glu were added into each well with a final concentration ranging from 2.5 to 100 μM, respectively, followed by 24 h of incubation. Cells were rinsed thrice with PBS before being exposed to H_2_O_2_ (600 μM) for 2 h of damage. A cell that was incubated without FA or FA-Glu, without the addition of H_2_O_2_ to induce oxidative damage, was used as a control. Cell viability was measured using an MTT assay.

#### 2.8.2. Intracellular ROS Evaluation

The concentration of ROS generated in PC12 cells was measured to investigate the antioxidant capacity of FA and FA-Glu. The PC12 cells were seeded in 12-well plates at a density of 3 × 10^5^ cells per well and incubated for 24 h. Cells were treated with FA and FA-Glu with gradient concentrations (20 μM, 40 μM and 100 μM) and further incubated for 24 h. After washing with PBS 3 times, the cells were exposed to H_2_O_2_ (600 μM) for 2 h. Cells were stained with 1:1000 diluted 2,7-dichlorofluorescein diacetate (DCFH-DA) in serum-free medium for 20 min, and the fluorescence intensity was measured using a flow cytometer (BD FACSCelesta, San Jose, CA, USA). Cells cultured without pre-treatment with FA or FA-Glu and oxidative damage by H_2_O_2_ were used as a control. Cells cultured without pre-treatment with FA or FA-Glu but damaged by H_2_O_2_ were used as a positive control.

Similarly, PC12 cells were treated with the above methods, followed by fixation in 4% paraformaldehyde for 30 min and washing with PBS three times. The fluorescence intensity was observed under a 20× laser scanning confocal microscope. 

### 2.9. Neuroprotective Effect Evaluation In Vivo

#### 2.9.1. Rat Model of Middle Cerebral Artery Occlusion (MCAO) and Treatments

Four rat groups (220 ± 20 g each) were randomly assigned: a sham operation, an ischemia–reperfusion (I/R), FA, and FA-Glu group (each n = 5). Prior to surgery, these groups were pre-treated with either saline, FA (i.p. 10 mg/Kg), or FA-Glu (i.p. 10 mg/Kg), respectively. Anesthesia was administered intraperitoneally using 10% chloral hydrate. The surgery was performed according to the protocol, involving a midline neck incision to expose the left common carotid artery (CCA), with aseptic insertion of a monofilament through the external carotid artery stump to obstruct the left middle cerebral artery (MCA) until slight resistance was felt. After 2 h of occlusion, the monofilament was removed for reperfusion. The sham group rats underwent the same surgical procedure but without MCA occlusion.

#### 2.9.2. Cerebral Infarction Volume Measurement Assay

Cerebral infarction volumes were evaluated after the staining of 2,3,5-triphenyltetrazolium chloride (TTC). Then, 24 h after MCAO, the rats were anesthetized and sacrificed via exsanguination by cardiac puncture. The brain tissues were isolated and frozen at −20 °C for 20 min, followed by coronal sections at 2 mm. The brain sections were stained with 2% TTC solution (G3005, Solarbio, Beijing, China) at 37 °C in the dark for 30 min and fixed in 4% paraformaldehyde for 24 h. The infarct volumes were quantified using ImageJ 1.53k (National Institutes of Health, Bethesda, MD, USA) and expressed as the percentage of infarction tissues relative to the sham group. The brain tissues were also stained using hematoxylin and eosin (H&E) for histological observation.

#### 2.9.3. MDA, GSH, SOD Measurements

The brain tissue levels of malondialdehyde (MDA), glutathione (GSH), and superoxide dismutase (SOD) were quantified using specific lipid peroxidation MDA assay, reduced glutathione assay, and total superoxide dismutase assay kits according to the manufacturers’ protocols, respectively. Each sample was assayed in triplicate for accuracy.

### 2.10. Statistical Analysis

All statistical analyses were performed using GraphPad Prism 8 software (GraphPad Software, San Diego, CA, USA). Data with normal distribution are presented as means ± standard deviation (SD). Data analysis was carried out using ANOVA with Tukey’s post hoc test. A *p* value < 0.05 was considered statistically significant.

## 3. Results and Discussion

### 3.1. Design and Synthesis of FA-Glu

The glycosylation design and synthesis of FA-Glu is important for achieving improved water solubility, stability, bioavailability, antioxidant activities, and stronger neuroprotection compared to FA. One such design is 4-*O*-β-D-glucopyranosyl FA. However, FA-Glu is specifically designed to conjugate glucose at the C6 position to FA at the 4-hydroxy position using 1,2,3-triazole as a linking bridge. The ability of the agent to cross the blood–brain barrier (BBB) remains a crucial factor and primary challenge in the development of central nervous system drugs [22]. A carrier-mediated transporter (CMT) system seems to be a promising method to facilitate the agent’s ability to cross the BBB. Glucose transporter 1 (GLUT1) is expressed in the endothelial cells, red blood cells, and astrocytes of the BBB, and is responsible for transmembrane glucose transport and providing the main energy source for brain tissue [23]. Based on the high affinity of glucose to GLUT1, the glucose–FA conjugate should penetrate the BBB easily. When glucose is conjugated with ibuprofen at the C6 position, its affinity with GLUT1 has minimal impairment [24]. This example supports the fact that the C6-OH linking position of glucose is appropriate for the GLUT1 target. Since 1,2,3-triazole is an ester group bioisostere in medicinal chemistry [25], it was used as the linker between FA and glucose to protect the FA phenolic hydroxyl group for better stability and bioactivity of the molecular structure. Additionally, 1,2,3-triazole can be constructed via the well-known “click” chemistry reaction under mild conditions. Therefore, the target derivative FA-Glu was designed and successfully synthesized.

FA was derived from compound **1** via propargylation at the C4-hydroxyl group. The yield was relatively low due to the esterification of COOH with 3-bromopropyne as a side reaction in the experimental conditions. The yield could be improved by increasing the mole ratio of 3-bromopropyne to promote as many reactions as possible, and hydrolyzing the ester by-products in stronger basic conditions. The alkyl group of compound **1** reacted with the azide group of compound **3**, and was catalyzed by Cu_2_SO_4_, to form a 1,2,3-triazole structure to produce the conjugate FA-Glu at an acceptable yield. The NMR data of compound **1** and FA-Glu are presented as follows.

Compound **1** (C_13_H_12_O_4_): ^1^H NMR (400 MHz, Chloroform-*d*): δ 7.65 (d, *J* = 15.9 Hz, 1H, CH=CHCOOH), 7.05 (d, *J* = 6.8 Hz, 1H, Benzene-5 H), 7.01 (s, 1H, Benzene-2 H), 6.90 (d, *J* = 8.2 Hz, 1H, Benzene-6 H), 6.29 (d, *J* = 15.9 Hz, 1H, CH=CHCOOH), 4.80 (d, *J* = 2.4 Hz, 2H, CH≡CCH_2_O), 3.89 (s, 3H, OCH_3_), 2.51 (s, 1H, C≡CH). ^13^C NMR (101 MHz, Chloroform-*d*) δ 166.43 (C=O), 148.32 (Benzene-4 C), 146.90 (Benzene-3 C), 146.12 (CH=CHCOOH), 126.77 (Benzene-1 C), 123.36 (Benzene-6 C), 114.89 (CH=CHCOOH), 114.31 (Benzene-5 C), 109.52 (Benzene-2 C), 77.99 (CH≡CCH_2_O), 74.95 (CH≡CCH_2_O), 55.99 (CH≡CCH_2_O), 52.00 (OCH_3_).

FA-Glu (C_19_H_23_N_3_O_9_): ^1^H NMR (400 MHz, Methanol-*d*_4_), δ 8.06 (s, 1H, *α* or *β*-NNNCH), 8.02 (s, 1H, *α* or *β*-NNNCH), 7.62 (d, *J* = 15.9 Hz, 1H, CH=**C**HCOOH), 7.16 (d, *J* = 1.7 Hz, 1H, Benzene-5 H), 7.05 (dd, *J* = 8.2, 1.7 Hz, 1H, Benzene-2 H), 6.78 (d, *J* = 8.2 Hz, 1H, Benzene-6 H), 6.34 (d, *J* = 15.9 Hz, 1H, CH=CHCOOH), 5.26 (s, 2H, NNNCCH**_2_**O), 4.84–4.74 (m, 2H, NNNCH**_2_**), 4.58–4.37 (m, 2H, Glucose-1 H, 2 H), 3.86 (s, 3H, OCH**_3_**), 3.74–3.59 (m, 1H, Glucose-5 H), 3.18–3.02 (m, 2H, Glucose-3 H,4 H). ^13^C NMR (101 MHz, Methanol-*d*_4_), δ 168.65 (C=O), 150.79 (Benzene-4 C), 149.37 (**C**H=CHCOOH), 147.41 (NNNCHCCH_2_O), 143.98 (Benzene-3 C), 127.56 (Benzene-1 C), 127.15 (Benzene-6 C), 124.20 (Benzene-5 C), 116.49 (NNNCHCCH_2_O), 114.88 (CH=CHCOOH), 111.78 (Benzene-2 C), 98.23 (Glucose-1 C), 93.99 (Glucose-1 C), 77.72 (Glucose-3 C), 76.06 (Glucose-3 C), 75.75 (Glucose-5 C), 74.61 (Glucose-5 C), 73.63 (Glucose-2 C), 73.10 (Glucose-2 C), 72.75 (Glucose-4 C), 71.22 (Glucose-4 C), 58.11 (NNNCHCCH_2_O), 56.45 (OCH_3_), 52.68 (NNNCH_2_), 52.59 (NNNCH_2_). Because there were α and β equilibrium configurations in the glucose pyran hemiacetal moiety of FA-Glu, this led to two important 1,2,3-trizaole alkene proton peaks at δ 8.06 and 8.02 in the ^1^H NMR spectrum, and six more pyran carbon peaks in the ^13^C NMR spectrum. LCMS-8045 (Shimadzu): Exact mass for C_19_H_23_N_3_O_9_ 437.1, found *m/z* [M + 1]^+^ 438.2.

The NMR and MS spectra can be seen in the Supporting Information.

### 3.2. Water Solubility and Stability

**Figure 1:**
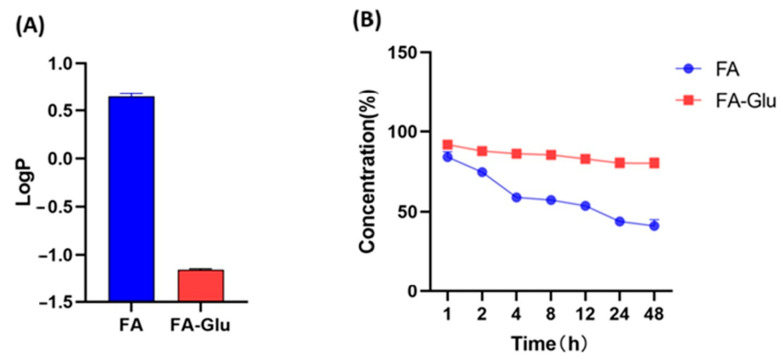
(**A**) LogP value of FA and FA-Glu (mean ± SD, n = 3). (**B**) Stability of FA and FA-Glu (mean ± SD, n = 3).

The LogP value measures the hydrophilicity or water solubility of a compound. As shown in Figure 1A, the LogP of FA was 0.64 ± 0.03, while the LogP of FA-Glu was much lower at −1.16 ± 0.01. The results demonstrated that FA-Glu possesses more polarity in its distribution in the water phase than FA. The introduction of the glucose moiety to FA increased the water solubility of the new FA-Glu conjugate, demonstrating its potential for better application and higher bioactivity.

FA undergoes degradation in the presence of light, heat, and alkaline pH. The introduction of 1,2,3-triazole could practically protect and improve the stability of FA’s molecular structure. The stability was measured via the change in the concentration percentage of FA-Glu and FA after they were incubated in PBS at different time points over a period of 48 h. As shown in Figure 1B, the concentration percentage of FA-Glu was 91.81% at 1 h, and it remained at 80.19% after 48 h, showing only slight degradation. Comparably, the concentration of FA was 84.05% at 1 h, but degraded to 41.24% after 48 h. The results indicate that the chemical stability of FA-Glu is better than that of its precursor FA.

### 3.3. Preventive Activity Against Oxidative Damage of pBR322 Plasmid DNA

**Figure 2:**
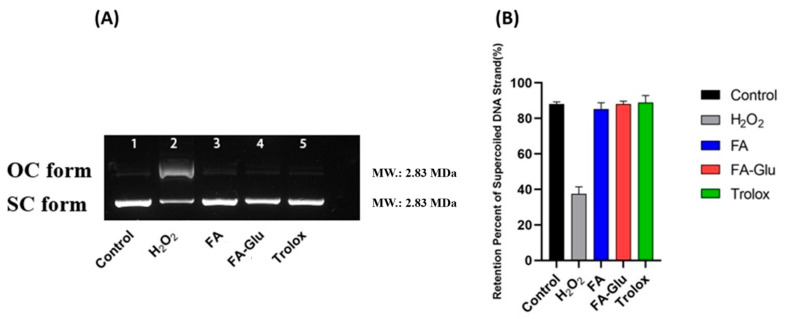
(**A**) Electrophoretic pattern of pBR322 plasmid DNA cleavage via •OH generated from the Fenton reaction and prevented by FA and FA-Glu. Lane 1: DNA + PBS (untreated control); lane 2: DNA + PBS + FeSO_4_ + H_2_O_2_; lane 3 and 4: DNA + PBS + FeSO_4_ + H_2_O_2_, in the presence of FA and FA-Glu, respectively; lane 5: DNA + PBS + FeSO_4_ + H_2_O_2_ + Trolox. n = 3. (**B**) Densitometric analysis of retention percentage of supercoiled DNA after damage induced by •OH generated from the Fenton reaction in the presence or absence of FA and FA-Glu (mean ± SD, n = 3).

Oxidative DNA damage in injured neurons is a common event during the early stages after cerebral ischemia [26,27,28]. The plasmid pBR322 is supercoiled (SC) circular double-stranded DNA. It transforms into a relaxing open circular (OC) form under oxidative stress. The oxidative protective effects of FA-Glu and FA against DNA damage were examined by monitoring their protection when converting plasmid pBR322 from the SC to OC form (Figure 2A) under oxidative stress. The SC form shows faster mobility than that of OC in the gel electrophoresis [29]. The retention percentage for the supercoiled DNA strand was calculated as SC% = SC/(SC + OC) × 100%. SC% represents the extent of plasmid DNA damage; a higher SC% value indicates less severe damage. As the results show (Figure 2), under oxidative stress conditions (DNA + PBS + FeSO_4_ + H_2_O_2_), SC plasmid DNA was cleaved into the OC form (Figure 2A lane 2). The other lanes, untreated control (lane 1, DNA + PBS), FA test lane 3, FA-Glu test lane 4, and Trolox control lane 5, were the main SC forms visualized. The results in Figure 2B quantitatively show that SC plasmid DNA underwent severe oxidative damage (with a low SC% value of 37.52%) via •OH, which was produced by the Fenton reaction (FeSO_4_ + H_2_O_2_), compared to the untreated control (88.05%). On the other hand, the FA test achieved an SC% value of 85.32%, while the FA-Glu test achieved a slightly higher value at 88.00%, comparable to that of the positive control Trolox test (89.05%). The SC% value results indicate that FA and FA-Glu efficiently protected against the oxidative damage to SC plasmid DNA using the •OH free radical. The introduction of glucose via 1,2,3-triazole did not change the ability of FA to scavenge the free radical. Since the H atom of FA 4-hydroxyl was substituted by the linker, FA-Glu could scavenge the hydroxyl radical reactions between the C=C bonds and •OH [30]. It is also probable that FA and FA-Glu reacted with H_2_O_2_ to restrict the Fenton reaction and the production of hydroxyl free radicals.

### 3.4. Cytotoxicity of FA-Glu

**Figure 3:**
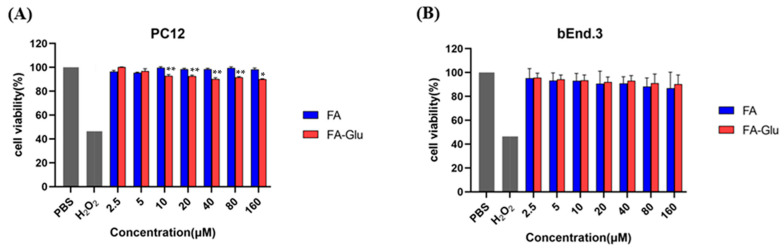
The cytotoxicity of FA and FA-Glu on PC12 cells (**A**) and bEnd.3 cells (**B**) (mean ± SD, *n* = 3; the same experiment was repeated thrice, * *p* < 0.05, ** *p* < 0.01 versus FA). bEnd.3 and PC12 cells without any treatment were used as control groups (cell viability 100%), respectively.

To investigate the safety of FA-Glu, a series of FA and FA-Glu concentrations were incubated with PC12 and bEnd.3 cells, respectively. Cytotoxicity was assessed using an MTT assay. As shown in Figure 3A, PC12 cell viability was 100% when treated with FA-Glu at 2.5 μM, followed by a slightly decreasing trend with increasing concentrations. Cell viabilities were almost stable at concentrations 10, 20, 40, and 80 μM of FA-Glu. It was 85.6% at the highest concentration of 160 μM of FA-Glu. When PC12 cells were treated with FA, all cell viabilities were above 90% at the test concentrations. At concentrations 10, 20, 40, 80, and 160 μM, PC12 cells treated with FA presented higher (* *p* < 0.05, or ** *p* < 0.01) viabilities than those treated with FA-Glu. The results show that FA-Glu exhibits a higher inhibition effect on PC12 cells than FA. In addition, all PC12 cell viabilities were above 80% from concentrations 2.5 to 160 μM, indicating that both FA and FA-Glu are non-toxic to the cell. The bEnd.3 cell is a reliable BBB model [31] and a BBB toxicity evaluation is necessary. The viability of the bEnd.3 cell is shown in Figure 3B. Both FA and FA-Glu groups showed slightly decreasing viability trends along with higher concentrations, with bEnd.3 cells treated with FA-Glu exhibiting slightly higher viabilities than cells treated with FA at all concentrations, though these differences were not significant. Perhaps the affinity of the glucose moiety to GLUT1 induced the higher safety levels of FA-Glu binding to bEnd.3 cell. The lowest cell viability was 82.6% with 160 μM FA; the lowest viability was 88.3% with the highest concentration of 160 μM FA-Glu. Both PC12 cell and bEnd.3 cell tests revealed FA-Glu to be a safe agent at test concentrations. The results provide appropriate concentration choices for the further evaluation of antioxidative effects.

### 3.5. FA-Glu Attenuate H_2_O_2_-Induced Antioxidative Injury on PC12 Cells

**Figure 4:**
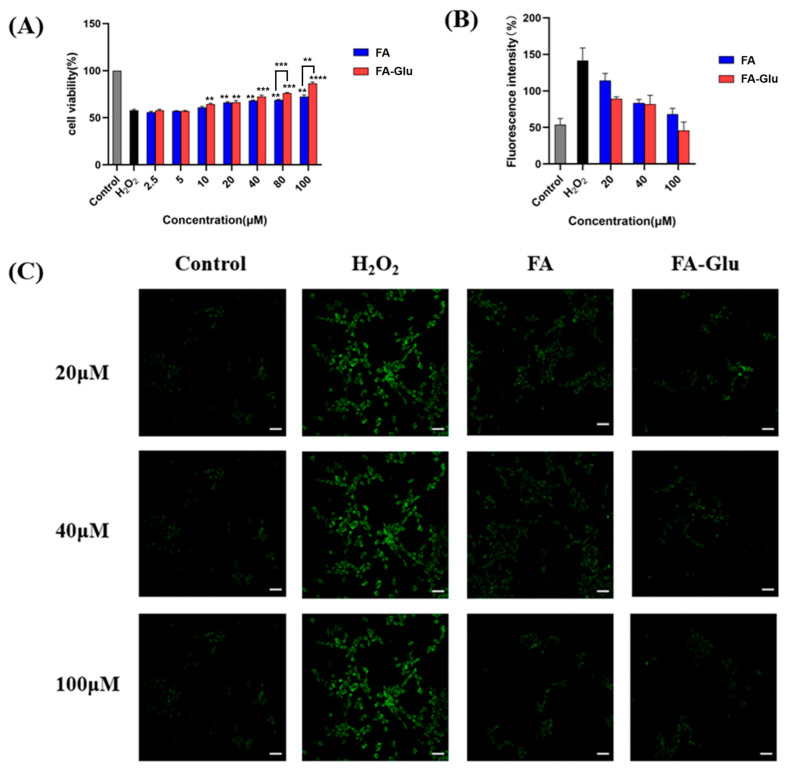
(**A**) PC12 cell viability, pre-treated with FA or FA-Glu, followed by H_2_O_2_ oxidative injury (mean ± SD, n = 3, ** *p* < 0.01, *** *p* < 0.001, **** *p* < 0.0001 versus H_2_O_2_). (**B**) PC12 ROS level, pre-treated with FA or FA-Glu, followed by H_2_O_2_ oxidative injury (mean ± SD, n = 3). (**C**) The ROS level of PC12 cells was measured using a confocal laser scanning microscope. The scale bar was 50 μm.

The PC12 cell in the logarithmic growth stage possesses typical neuronal morphology and function [32]. It is a reliable neuronal cell model. To examine FA-Glu’s protection effects against oxidative damage in neuronal cells, the PC12 cell was incubated with FA and FA-Glu at concentrations of 2.5, 5, 10, 20, 40, 80, and 100 μM, respectively. H_2_O_2_ was added to induce oxidative cell damage, followed by the MTT assay to assess cell survival rates. Cell viability is shown in Figure 4A. Compared to the control (treated with PBS, with 100% cell viability), PC12 cells that were not co-incubated with FA or FA-Glu underwent significant oxidative damage via H_2_O_2_, with cell viability decreasing to 57.8% (positive control). This established the H_2_O_2_-induced nerve injury model. For cells co-incubated with FA or FA-Glu at low concentrations (2.5 and 5 μM), the cell damage was similar to that of the positive control, with no antioxidative protection found from the agents. Starting at 10 μM, FA-Glu exhibited reduced cell oxidative damage effects compared with the positive control (*p* < 0.01). At concentrations of 20 and 40 μM, both agents significantly attenuated oxidative cell damage. When the concentration was 80 μM, cells co-incubated with FA-Glu survived (*p* < 0.001) with 76.3% cell viability, which was higher than cells co-incubated with FA (*p* < 0.001). The most significant antioxidant effects of FA-Glu were identified at a concentration of 100 μM (*p* < 0.0001) with 86.6% cell viability, higher than FA (*p* < 0.01). At all test concentrations using FA, cell viabilities were below 80%. The results demonstrate that FA-Glu is superior to FA, effectively suppressing H_2_O_2_-induced oxidative injury in PC12 cells with increasing concentrations, thus benefiting neuronal protection.

### 3.6. FA-Glu Inhibition of ROS Production in the Oxidative Injury of PC12 Cells

For more insight into the antioxidant property of FA-Glu, its ability to inhibit ROS production in the oxidative injury of PC12 cells was quantitatively examined using the DCFH-DA method with a flow cytometer. As Figure 4B shows, ROS production levels decreased as the FA or FA-Glu concentration increased. FA-Glu significantly inhibited the ROS production induced by H_2_O_2_ oxidation. Specifically, when the cells were pre-treated with the agent at 100 μM, the ROS level was reduced to 45.67%, which was comparable to that of the control group. Conversely, FA-Glu completely prevented the additional ROS production induced by H_2_O_2_ at this concentration. The ROS result was consistent with the results in Section 3.5, with the highest cell viability achieved at 100 μM. In contrast, FA exhibited a moderate capacity for preventing ROS production. Its ROS levels were higher than those of FA-Glu at all tested concentrations. The results show that FA has a lower capability to attenuate ROS generation than FA-Glu.

The results of the confocal qualitative study are shown in Figure 4C. The green fluorescence intensity represents the ROS concentration. Lower fluorescence intensity meant lower ROS generation levels in the cells. The positive control (H_2_O_2_) image is clear and bright, while those of the FA and FA-Glu groups show very faint observations. The image of the FA-Glu group at 100 μM was the weakest and closely matched the control. The confocal image results are consistent with the flow cytometer results and cell viability results in Section 3.5. The PC12 cell results demonstrate that FA-Glu can protect neuronal cells from oxidative injury by preventing ROS production in the cells. Overall, FA-Glu is superior to its precursor FA regarding antioxidant activities.

To provide the mechanistic basis of neuroprotection, investigations into the ability of FA-Glu to alter its antioxidant enzymes (e.g., SOD, GPx) and/or makers of apoptosis (e.g., Bcl-2, Bax, cleaved caspase-3) in the PC12 model should be carried out. This is a limitation of current PC12 cell tests.

### 3.7. Neuroprotective Effects of FA-Glu on Cerebral Ischemia–Reperfusion

**Figure 5:**
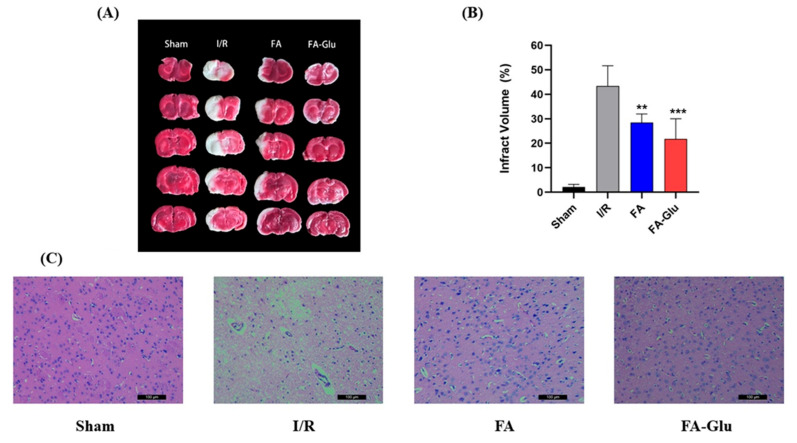
(**A**) Brain sections with TTC staining (red color denotes normal brain sections; white color indicates infarcted area). (**B**) Brain tissue infarction percentage relative to that of the sham group via TTC staining (mean ± SD, n = 5; ** *p* < 0.01, *** *p* < 0.001 versus I/R group). (**C**) Brain sections with H&E staining, scale bar 100 μm.

The neuroprotective effects of FA and FA-Glu were further evaluated, in vivo, using a rat model of middle cerebral artery occlusion (MCAO). The TTC staining of brain sections are shown in Figure 5A,B. Red denotes normal brain sections, while white indicates infarcted brain areas. The percentage of cerebral ischemia in the sham group was 2.2%, showing that the MCAO model was well-established. The infarcted area percentage reached 43.42% in the ischemia–reperfusion (I/R) group, indicating the successful establishment of the I/R model. The percentage was 28.49% for the FA group. This downward trend is reflected by the 21.79% of the FA-Glu group. Both FA (*p* < 0.01) and FA-Glu (*p* < 0.001) significantly reduced the infarcted brain areas compared to the I/R group, with FA-Glu showing greater neuroprotective activity than FA. These findings were consistent with that of PC12 cell oxidative damage experiments.

The H&E staining of brain sections reflected brain histological injury, as shown in Figure 5C. In the sham group, cells were retained intact and formed closely arranged structures. Nuclei were clearly stained. On the contrary, cell structure was damaged in the I/R group as vacuoles were found and cytoplasm was stained. H&E staining results also confirmed the establishment of the I/R model. The FA and FA-Glu groups showed significant improvements in histological features, though the FA-Glu group was still better than the FA group. The cell arrangement in the FA-Glu group was more compact, and the cell status was similar to that of the sham group, exhibiting a tight cell arrangement, uniform staining, and a good overall condition. Consequently, pre-treatment with FA-Glu could improve the oxidative damage induced by CIRI. This result is consistent with the findings from the TTC staining experiment.

**Figure 6:**
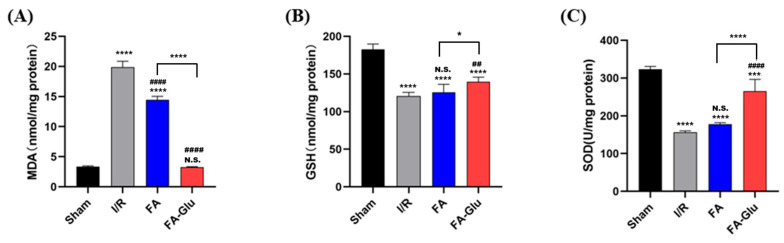
(**A**) MDA level. N.S.: no significant difference between FA-Glu group and sham group. (**B**) GSH level. N.S.: no significant difference between FA group and I/R group. (**C**) SOD enzyme activity. N.S.: no significant difference between FA group and I/R group. (mean ± SD, n = 5). * *p* < 0.05, *** *p* < 0.001, **** *p* < 0.0001 versus sham group; ^##^ *p* < 0.01, ^####^ *p* < 0.0001 versus I/R group.

The oxidative stress markers, i.e., MDA, GSH, and SOD, in the brain tissues were also measured. The results are shown in Figure 6. MDA is the final product of lipid peroxidation. MDA promotes the oxidative modification of proteins, altering their structural and functional properties [33]. As such, MDA is an important parameter reflecting the potential antioxidant capacity of the body. In this measurement, the MDA level was excessive at 19.86 nmol/mg of protein in the I/R group. The FA group’s MDA level decreased to 14.45 nmol/mg of protein, versus the I/R group (*p* < 0.0001). Similarly, FA-Glu sharply reduced the MDA level to 3.26 nmol/mg of protein, compared to the I/R group (*p* < 0.0001). The MDA level of the FA-Glu group was comparable to that of the sham group. FA-Glu almost reversed the MDA level in the MCAO experiments. Relevant to this, GSH is important in maintaining cellular redox homeostasis, and it can effectively eliminate free radicals and ROS to prevent cellular damage and dysfunction [34]. The GSH content in the I/R group was significantly (*p* < 0.0001, versus sham group) decreased to 120.7 nmol/mg of protein, similar to the decreasing trend experienced by the FA group to 125.8 nmol/mg of protein. Yet the GSH content in the FA-Glu group increased significantly (*p* < 0.01) to 139.6 nmol/mg protein, compared to the I /R group. Since SOD scavenges free radicals and plays a role in cellular self-defense mechanisms against oxidative stress [35], it is recognized as another important oxidative marker. A similar trend to that of GSH was observed for the SOD measurement results. The SOD level in the FA group increased slightly compared to the I/R group’s 156.5 U/mg of protein. FA-Glu could significantly increase (*p* < 0.0001) brain SOD levels to 265.19 U/mg of protein, compared to the I/R group. The results of three oxidative stress markers suggest that FA-Glu can alleviate oxidative damage by decreasing the MDA level and increasing the GSH level and SOD enzyme activity. The cerebral ischemia–reperfusion results demonstrated that FA-Glu can exert antioxidative neuroprotection effects, making it superior to FA precursor.

The CIRI model study was limited as nimodipine or edaravone was not used as positive controls. Thus far, FA-Glu’s effects on inflammatory (e.g., IL-1β, TNF-α) or neuronal damage markers (e.g., NeuN, GFAP) have not been investigated. Measurement of FA-Glu effects on these markers will contribute the mechanistic data and support the therapeutic potential of FA-Glu.

FA-Glu was derived from FA glycosylation through the 1,2,3-triazole linker. All results were in line with the molecule design. FA-Glu showed greater water solubility and chemical stability than FA in this study. Generally, 1,2,3-triazole is stable to acidic or basic hydrolysis, metabolic degradation, and redox condition [36]. The triazole linker supported FA-Glu stability. Glucose was conjugated to FA via the C6 position. The glucose moiety of FA-Glu not only led to higher solubility but also resulted in FA-Glu’s higher affinity and crossing ability to the BBB mediated by GLUT1. FA-Glu showed greater neuroprotective activity than FA. It is possible that FA-Glu had a higher distribution in brain tissue and exerted antioxidative activity due to its molecular structure. FA-Glu is a possible free radical scavenger and inhibitor of enzymes that catalyze the production of free radicals, an enhancer of enzyme activity for scavengers, and an inhibitor of inflammation. These possibilities could be clarified via further mechanisms, bioavailability, and pharmacokinetic studies.

## 4. Conclusions

The novel FA derivative FA-Glu was efficiently prepared. By comparison, FA-Glu had higher water solubility and chemical stability, and could prevent pBR322 plasmid DNA cleavage induced by •OH radicals, similar to FA. FA-Glu is a safe agent based on PC12 and bEnd.3 cell tests, and is comparable to FA. The PC12 cell test indicated that FA-Glu significantly prevented H_2_O_2_-induced oxidative damage and inhibited excessive ROS generation in the cell. In vivo animal model MCAO evaluations consistently demonstrated that FA-Flu can significantly reduce infarcted brain areas using the measurements of TTC stain observations, quantification, and the histological evaluation of H&E stains, and was superior to FA. The measurements of oxidative stress markers in the brain tissues, including MDA, GSH, and SOD levels, were all consistent with the above results. In summary, FA-Glu increased its precursor’s physical and chemical properties, exhibited better of antioxidative neuroprotective effects, and alleviated cerebral ischemia–reperfusion injury (CIRI). The results of evaluating FA-Glu surpass those of FA. One of the reasons for this could be attributed to glucose’s (C6 position conjugate) affinity for the GLUT1 receptor on the BBB and neuronal cells, enabling delivery to the brain. FA-Glu has therapeutic potential for IS and other neuronal problems, which should be the subject of further research.

## Data Availability

The original contributions presented in this study are included in the article/Supplementary Material. Further inquiries can be directed to the corresponding author(s).

## Supplementary Materials

The following supporting information can be downloaded at: https://www.mdpi.com/article/10.3390/antiox14080953/s1: NMR spectra of compound **1** and FA-GLu, MS spectrum of FA-Glu.
