# Supplementary figures and images for: A Novel Glycosylated Ferulic Acid Conjugate: Synthesis, Antioxidative Neuroprotection Activities In Vitro, and Alleviation of Cerebral Ischemia–Reperfusion Injury (CIRI) In Vivo
Source: Antioxidants (Basel). 2025 Aug 3;14(8):953. doi: 10.3390/antiox14080953 (PMC12382698; doi:10.3390/antiox14080953)

# MS of FA-Glu.

LCMS-8045 (Shimadzu): Exact mass for  $C_{19}H_{23}N_3O_9$  437.1, found  $[M+1]^+$  438.2.

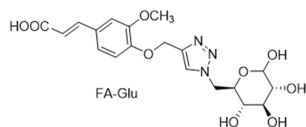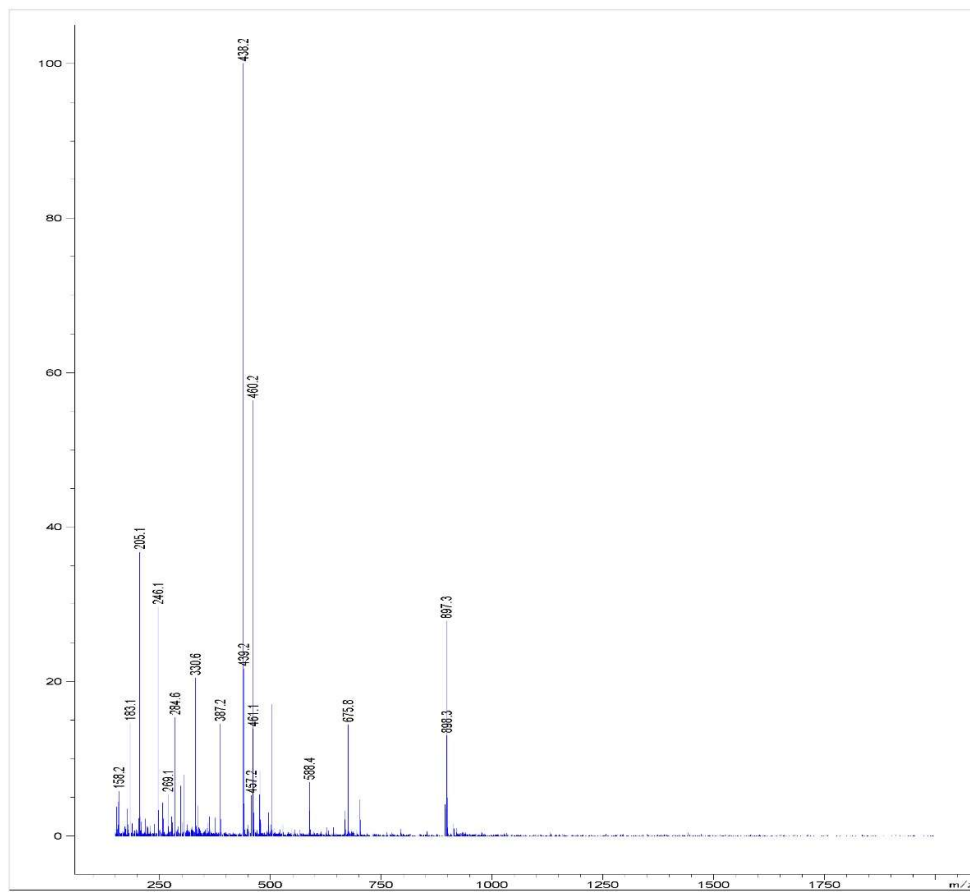

Supplement: Supplementary file 1 [file antioxidants-14-00953-s001.zip › antioxidants-3738180-supplementary.pdf]
